# A Genome Doubling Event Reshapes Rice Morphology and Products by Modulating Chromatin Signatures and Gene Expression Profiling

**DOI:** 10.1186/s12284-021-00515-7

**Published:** 2021-08-04

**Authors:** Chao Zhou, Xiaoyun Liu, Xinglei Li, Hanlin Zhou, Sijia Wang, Zhu Yuan, Yonghong Zhang, Sanhe Li, Aiqing You, Lei Zhou, Zhengquan He

**Affiliations:** 1 Key Laboratory of Three Gorges Regional Plant Genetics and Germplasm Enhancement (CTGU), Biotechnology Research Center, China Three Gorges University, Yichang, 443002 China; 2 Institute for Interdisciplinary Research, Jianghan University, Wuhan, 430056 China; 3 Bioacme Biotechnology Co., Ltd., Wuhan, 430056 China; 4 Hubei Key Laboratory of Wudang Local Chinese Medicine Research, Hubei University of Medicine, Shiyan, 442000 China; 5 Hubei Key Laboratory of Food Crop Germplasm and Genetic Improvement, Food Crops Institute, Hubei Academy of Agricultural Sciences, Wuhan, 430064 China

**Keywords:** Rice, Autopolyploid, Accessible chromatin regions, Transcriptional regulation, Epigenetic marks

## Abstract

Evolutionarily, polyploidy represents a smart method for adjusting agronomically important in crops through impacts on genomic abundance and chromatin condensation. Autopolyploids have a relatively concise genetic background with great diversity and provide an ideal system to understand genetic and epigenetic mechanisms attributed to the genome-dosage effect. However, whether and how genome duplication events during autopolyploidization impact chromatin signatures are less understood in crops. To address it, we generated an autotetraploid rice line from a diploid progenitor, *Oryza sativa* ssp. *indica* 93-11. Using transposase-accessible chromatin sequencing, we found that autopolyploids lead to a higher number of accessible chromatin regions (ACRs) in euchromatin, most of which encode protein-coding genes. As expected, the profiling of ACR densities supported that the effect of ACRs on transcriptional gene activities relies on their positions in the rice genome, regardless of genome doubling. However, we noticed that genome duplication favors genic ACRs as the main drivers of transcriptional changes. In addition, we probed intricate crosstalk among various kinds of epigenetic marks and expression patterns of ACR-associated gene expression in both diploid and autotetraploid rice plants by integrating multiple-omics analyses, including chromatin immunoprecipitation sequencing and RNA-seq. Our data suggested that the combination of H3K36me2 and H3K36me3 may be associated with dynamic perturbation of ACRs introduced by autopolyploidization. As a consequence, we found that numerous metabolites were stimulated by genome doubling. Collectively, our findings suggest that autotetraploids reshape rice morphology and products by modulating chromatin signatures and transcriptional profiling, resulting in a pragmatic means of crop genetic improvement.

**Supplementary Information:**

The online version contains supplementary material available at 10.1186/s12284-021-00515-7.

## Background

Rice is one of the most important food crops in the world and provides more than 20% of the caloric intake for one-half of the world’s population (Zhang et al. 2016). Polyploidy, or whole-genome duplication (WGD), is an effective method to increase the size of the rice genome and improve its adaptability (Tu et al. 2014; Yu et al. 2020). In general, there are two major categories of polyploidy in plants. Allopolyploids inherit different subgenomes after interspecific hybridization, whereas the subgenomes of autopolyploids arise from the same species (Wendel 2000; Otto 2007). Allopolyploidy is thought to have played a significant role in the long-term evolution of plants and remains an important speciation process (Soltis et al. 2009; Abbott et al. 2013). There are numerous studies dealing with genetic and epigenetic changes associated with polyploidization in newly created allopolyploid materials (Lukens et al. 2006; Wang et al. 2006; Flagel and Wendel 2010; Rapp et al. 2009). However, increasing evidence indicates that the real appearance of autotetraploid plants in nature might be significantly underestimated. It is widely known that, doubling an entire set of chromosomes in vivo may also induce a series of problems, such as gene-expression aberrations, which could be detrimental challenges to newly formed polyploidy (Madlung and Wendel 2013). To better understand how natural selection enables neopolyploidy to overcome early challenges is an urgent issue for biologists. Given that allopolyploids can be confounded by the entanglement of both WGD and hybridization, synthesized autopolyploid systems are ideal systems to investigate the short-term effects produced by WGDs, such as those in *Arabidopsis* (Zhang et al. 2019), rice (Chen et al. 2019; Wu et al. 2017; Yu et al. 2020; Guo et al. 2017; Wang et al. 2021), potato (Stupar et al. 2007), *Citrus limonia* (Allario et al. 2011) and willows (Dudits et al. 2016). To some extent, according to these results, it is possible that the change in transcriptional activity induced by autotetraploid plants could be attributed to nuclear dosage (Riddle et al. 2010; Stupar et al. 2007; Allario et al. 2011). However, to explore the mechanisms controlling gene transcription changes induced by genome duplication, further investigations are still needed.

Recent studies have illustrated that differences in chromatin accessibility are key factors in the chromosome-scale patterns of gene expression changes seen in dosage compensation (Giorgetti et al. 2016) and in large-scale transcriptional reprogramming (Miyamoto et al. 2018). Thus, chromatin accessibility changes may provide new insight into the intrinsic nature of the regulation of transcription after plant autopolyploidization. Continuous efforts have focused on studying chromatin accessibility in plant allopolyploids (Jordan et al. 2020; Lu et al. 2020), implying that chromatin accessibility influences differential gene expression induced by allopolyploidization. However, characterization of open chromatin and investigation of their biological effects in autopolyploidization are lacking.

Accessible chromatin regions provide physical scaffolds to recruit transcriptional co-regulators and displace their nearby nucleosomes in multiple plant species (Lu et al. 2019; Zhou et al. 2020). Most recently, transposase-accessible chromatin sequencing (ATAC-seq), which relies on an engineered Tn5 transposase to cleave accessible DNA regions, has been developed as a reliable tool for profiling ACRs across multiple plant species and cell types (Buenrostro et al. 2013, 2015). Applications of ATAC-seq have been successfully utilized in rice (Wilkins et al. 2016; Yang et al. 2020), *Arabidopsis thaliana* (Lu et al. 2017; Sijacic et al. 2018), *Medicago truncatula* (Maher et al. 2018), *Sorghum bicolor* (Zhou et al. 2020) and others (Lu et al. 2019). These results demonstrate that ACRs are prevalent and have highly dynamic functions in distinct chromatin pathways that regulate gene expression in plants. In addition, a substantial number of open chromatin regions have been identified through a set of comparative genomic and epigenomic analyses in 13 plant species, including rice (Lu et al. 2019). Consequently, genic ACRs (gACRs) and proximal ACRs (pACRs) are enriched with particular active transcription-associated histone modifications, including H3K4me3, H3K56ac, and H3K36me3 (Lu et al. 2019). In contrast, distal ACRs (dACRs) can be in an unmodified state, an H3K56ac-modified state, which is associated with the active transcription of nearby genes as an enhancer, or a H3K27me3-modified state, which is probably involved in Polycomb group (PcG) silencing pathways as a repressor (Lu et al. 2019). Consistently, enhancers harboring dACRs in rice are always characterized by H3K27me3 and H3K4me3 and/or H3K27ac (Sun et al. 2019). In the maize genome, it has been shown that ACRs are associated with H3K9ac and H3K27ac, but not with H3K4me1 (Lu et al. 2019; Ricci et al. 2019). Long-range chromatin loops accumulating between dACRs or between dACRs and genes can control transcriptional activity (Ricci et al. 2019). Thus, the identification of distinct chromatin features using ATAC-seq coupled strategies will provide novel genomic and epigenomic insights into how chromatin pathways regulate plant gene expression during autopolyploidization.

Here, we combined ATAC-seq, RNA-seq, and metabolomics data approaches to explore whether significant ACR alterations that occur in response to genome doubling can be found by comparing synthesized autotetraploid rice with its parent diploid. The results of this study provide a comprehensive map of WGD-induced dynamic assessable regions in the autotetraploid rice genome. We observed that the euchromatin, but not the heterochromatin, of the autopolyploid genome becomes more accessible. Our findings indicated that the functional regulation of genic ACR on transcription was enhanced in the process of rice genome doubling. In particular, the combination of H3K36me2 and H3K36me3 may be associated with chromatin accessibility in rice autopolyploidization. We also found that metabolite accumulation could be used as a biomarker for plant genome duplication. These results shed light on chromatin accessibility coupled with histone marks and transcriptional regulation in autotetraploid plants.

## Materials and Methods

### Plant Materials and Growing Conditions

The rice (*O. sativa* ssp. *indica*) plants used in this study were from the ‘93–11’ background. According to previously published methods (Raina and Irfan 1998), we derived autotetraploid rice from anther cultures of diploid rice. Rice materials were independently self-pollinated over 10 generations at the Hubei Academy of Agricultural Sciences (Wuhan China). Seedlings were grown in one-half-strength Murashige and Skoog medium under 16-h light/8-h dark cycles at 30 °C. Leaves of 12-day-old seedlings were harvested and frozen in liquid nitrogen before follow-up experiments.

### Flow Cytometry

Autotetraploid and diploid seedlings were collected into clean and precooled plates and then chopped with a fresh razor blade to release the nuclei in sterile lysis buffer (100 mM citric acid, 0.5% Tween 20, pH 2.0–3.0, 400 mM Na_2_HPO_4_·12H_2_O) for 3–5 min until the buffer turned green. The mixture was transferred into a 40 μm strainer. DAPI solution was added to the filtrates at a final concentration of 1 ng/ml and then incubated for 30 min in the dark on ice. The DAPI-treated filtrates were then loaded on a flow cytometer (Beckman Coulter CytoFLEX S, USA) to measure the ploidy levels. We thank Shuyan Liang and Zhixin Qiu from Wuhan Biobank Co., Ltd. for their kind help in the flow cytometric analysis of experimental samples.

### ATAC-seq and Analysis

Frozen samples for ATAC were obtained according to the ATAC-seq protocol from Shanghai Jiayin Biotechnology Ltd. (Shanghai). In brief, native nuclei were purified from frozen samples as previously described (Corces et al. 2017). The Nextera DNA Library Preparation Kit (Illumina) was used to perform transposition according to the manufacturer’s manual. Fifty thousand nuclei were pelleted and resuspended in transposase for 30 min at 37 °C. The transposed DNA fragments were purified immediately using a MinElute PCR Purification Kit (Qiagen). After that, samples were PCR-amplified using 1 × NEBNext High-Fidelity PCR Master Mix (New England Biolabs, MA). Subsequent libraries were purified with the MinElute PCR Purification Kit (Qiagen) and subjected to sequencing on an Illumina NovaSeq 6000 using PE150.

Raw reads were trimmed using Trim_galore with the following filter parameters: ‘-q 25 –phred33 –length 35 -e 0.1 –stringency 4’ (https://github.com/FelixKrueger/TrimGalore). The 93–11 reference genome was obtained (Zhang et al. 2018). Clean reads were aligned to the reference genome using Bowtie v.1.1.2 (Langmead et al. 2009) with the following parameters: ‘bowtie -X 1000 -m 1 -v 2 –best –strata’. Aligned reads were sorted using Samtools v.1.3.1 (Li et al. 2009). Clonal duplicates were removed using Picard v.2.16.0 (http://broadinstitute.github.io/picard/).

### ChIP-seq and Data Analysis

The ChIP experiment was performed as described (Zhou et al. 2021). About 2 g of 12-day-old of seedling leaves was cross-linked in 1% formaldehyde under vacuum. Chromatin was extracted and fragmented to 200–500 bp by sonication, and ChIP was performed using the following antibodies: anti-H3K36me2 (ab9049) and anti-H3K36me3 (ab9050). DNA from ChIP was used to construct sequencing libraries following the protocol provided by Illumina TruSeq® ChIP Sample Prep Set A. The library construction and sequencing were completed at Bioacme Biotechnology Co., Ltd. and the library products were sequenced with the Illumina HiSeq-PE150 platform.

Low-quality reads were removed from raw data using the Trimmomatic package (version0.35) (http://www.usadellab.org/cms/uploads/supplementary/ Trimmomatic/Trimmomatic-Src-0.35.zip) (Bolger et al. 2014), and the clean data were mapped to mapping to the 93–11 reference genome (Zhang et al. 2018) using BWA (Li and Durbin 2009) with parameters permitting less than 2 mismatch. Then, Samtools (version 0.1.19) (Li et al. 2009) was used to remove potential PCR duplicates and model-based analysis for chromatin immunoprecipitation sequencing (ChIP-seq) (MACS) software (version 1.4.2) (Zhang et al. 2008) was introduced to locate enriched regions to call peaks by comparing reads from the IP sample with the input sample. Wig files produced by MACS software were used for data visualization by IGV (version 2.3.88) (Robinson et al. 2011). DeepTools 2.0 (Ramírez et al. 2016) software was used to generate a heatmap of different epi-modifications.

### RNA-seq Analysis

Total RNA (10 mg) was used to purify poly (A) mRNA. mRNA was used for the synthesis and amplification of complementary DNA. The RNA-seq libraries were prepared using the TruSeq RNA Sample Preparation Kit from Illumina. Libraries were sequenced on an Illumina HiSeq. The experiments, including library construction, and sequencing were performed at Annoroad Gene Technology Co. Ltd (Beijing). RNA-seq data were first cleaned by removing contaminations and low-quality reads by Trimmomatic (Bolger et al. 2014). Subsequently, two analysis pipelines were performed. As a first approach, the clean data were aligned with TopHat 2.0 (Kim et al. 2013) using the 93–11 rice genome as the reference under default parameters. Then, the FPKM values were calculated with Cufflinks, and the differentially expressed genes (log [fold change] > 1, *P* < 0.05) were calculated by Cuffdiff (Trapnell et al. 2010). An alternative to the steps above is to map clean data to 93–11 rice genome with Hisat2 (Kim et al. 2015), and calculate integer read count with featureCounts (Liao et al. 2014). According to a previous method (Zhang et al. 2019), the DEGs analysis was run using the classical normalization with DEseq2 (Love et al. 2014) and EdgeR (Robinson et al. 2010) with a 0.05 *P* value and cutoff twofold change.

### Identification of ACRs

The process of calling ACRs was conducted according to previously described protocols, with slight modifications (Lu et al. 2019; Zhou et al. 2020). MACS2 was used to define ACRs with the ‘– keepdup all’ function. To find high-quality ACRs, the following filtering steps were generally performed: (1) peaks called with MACS2 were split into 50 bp windows with 25 bp steps; (2) the Tn5 integration frequency in each window was calculated and normalized to the average frequency in the total genome; (3) windows passing the integration frequency cutoff were merged together with 150 bp gaps; (4) small regions with only one window were then filtered with ‘length > 50 bp’. The sites within ACRs with the highest Tn5 integration frequency were defined as summits (Ricci et al. 2019; Zhou et al. 2020).

### Gowinda Analysis

The Gowinda tool was applied for functional enrichment analysis of the selected gene lists, with default parameters (Kofler and Schlötterer 2012).

### HPLC-Q-TOF/MS Analysis

The rice samples were ground into fine powder with liquid nitrogen. Rice powder (100 mg) was placed in a 1.5 mL centrifuge tube, and then 400 μL of 75% (v/v) methanol containing 0.1% formic acid (v/v) was added. After vortexing for 30 s and sonicating at 20 °C for 15 min, the sample was subsequently mixed for 2 min by vortexing and centrifuged at 12,000 rpm for 20 min and 4 °C. The supernatant was collected for analysis by HPLC-Q-TOF/MS. All samples were analyzed by high-performance liquid chromatography combined with a quadrupole time-of-flight mass spectrometry (HPLC-Q-TOF/MS, 6545) system. An Agilent Zorbax Eclipse Plus C18 column (2.1 mm × 100 mm, 1.8 μm) was used to separate the extracted compounds. Analyses were separated by gradient elution with ultrapure water (A) and acetonitrile (B), both containing 0.1% formic acid (v/v) at a flow rate of 0.30 mL/min. The gradient was as follows: 0–2 min, 5% B; 2–20 min, 5–100% B; and 20–25 min, 100% B. The post run time was 5 min. The column temperature was set to 40 °C with an injection volume of 2 μL. An Agilent 6545 ESI-Q-TOF system from Agilent Technologies was applied for MS acquisition. Both positive and negative ionization modes were performed. Mass spectrometry parameters were set as follows: capillary voltage of 4.0 kV (positive mode) and 3.5 kV (negative mode), a gas temperature of 325 °C, a drying gas flow rate of 8 L/min, a nebulizer pressure of 35 psi, a sheath gas temperature of 360 °C with a gas flow rate of 12 L/min, and a mass range of *m*/*z* 100–1700. Agilent profinder software was used for retention time correction, peak integration and alignment. After the CEF file output, Agilent Mass Profiler Professional software was applied for statistical processing, and the METLIN database was used for metabolite identification. The experiments were performed by Bioacme Biotechnology Co., Ltd. (Wuhan, China).

### DNase I Digestion and PCR Detection

Frozen rice leaf powder was suspended in 10 mL of ice-cold nuclei isolation buffer (1 M hexylene glycol, 20 mM PIPES-KOH, pH 7.6, 10 mM MgCl_2_, 1 mM EGTA, 15 mM NaCl, 0.5 mM spermidine, 0.15 mM spermine, 0.5% Triton X-100 [v/v], 10 mM β-mercaptoethanol, and 1 × protease inhibitor cocktail [Roche]). The mixture was incubated for 15 min at 4 °C with gentle shaking. Nuclei extracts were washed once with 1 mL of digestion buffer (40 mM Tris–HCl, pH 7.9, 0.3 M Sucrose, 10 mM MgSO_4_, 1 mM CaCl_2_, and 1 × protease inhibitor cocktail [Roche]) and gently resuspended on ice in fresh digestion buffer by pipetting until no clumps were visible. A DNase I (RQ1 RNase-Free DNase; Promega) dilution series was prepared by stepwise dilution using digestion buffer and kept on ice. For the digestion of isolated DNA, genomic DNA was extracted from the nuclear suspension using phenol:chloroform:isoamyl alcohol extraction and ethanol precipitation. Then, the extracted DNA was digested with DNase I at final concentrations of 0, 0.25 and 0.5 units mL^−1^. Finally, chromatin DNA and genomic DNA were detected with primers (Additional file 9: Table S8).

## Results

### Morphological Changes Caused by Rice Genome Doubling

**Fig. 1:**
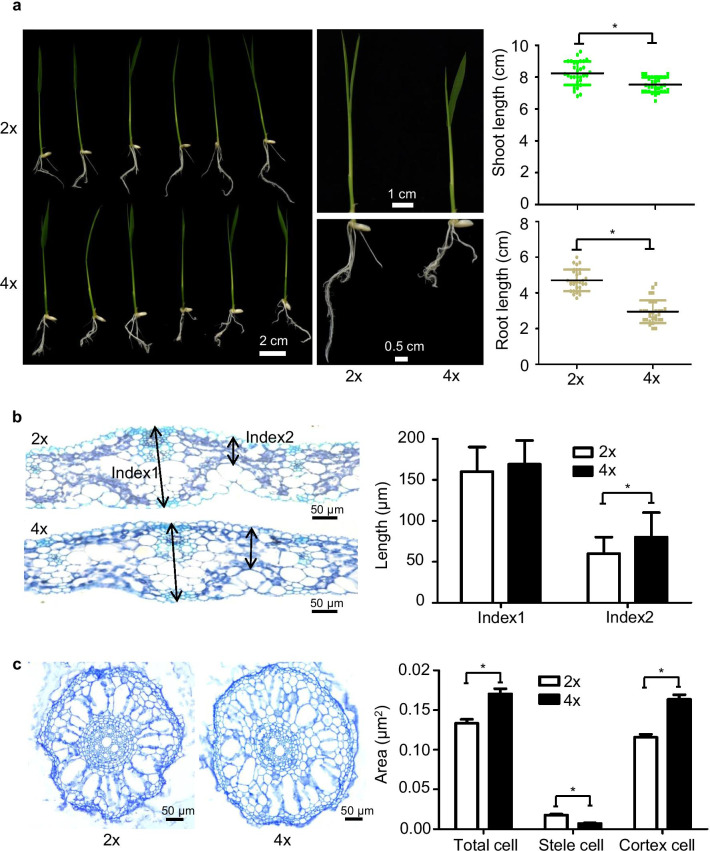
Distinct phenotypes of autotetraploid rice compared with its diploid parent. **a** Seedling morphology of diploid and autotetraploid rice. Phenotype of 2 × and 4 × rice at 10 days after germination (left). Leaf (green) and root (yellow) length measurements are shown on the right. *P < 0.05, *t* test, two-sided (*N* = 30). **b** Light micrographs of cross-sections from 2 × and 4 × leaves. The cell layers of the cross sections (indicated as index1 and index2) through the leaf midvein were measured and statistically calculated by *t* test. Error bar represents SD (*N* > 50). Bar = 50 μm. **c** Cross-sections from 2 × and 4 × rice roots. Scale bars are 50 μm. Statistical analysis of three types of root cells, including stele cells, cortex cells, and total cells, is shown (right), *P < 0.05, two-sided *t* test (*N* > 45)

To study the impact of WGD without interspecies hybridization effects, we induced autotetraploid rice (4x) from a diploid cultivar (2x), *Oryza sativa* ssp. *indica* cv. 93–11, which had been independently self-pollinated over 10 generations. Diploid and autotetraploid rice were confirmed by flow cytometry (Additional file 1: Figure S1). The autotetraploid rice exhibited remarkable differences in morphological traits compared with the diploid rice, such as decreased leaf size, root length and whole plant size during dynamic growth (Fig. 1a and Additional file 1: Figure S2a). Moreover, at the 12-day seedling stage, a significantly thicker leaf midvein (Fig. 1b) and greater cortex cell area (Fig. 1c) were observed in 4 × rice. At mature stages, the 4 × rice displayed enlarged plant height and panicle and grain sizes (Additional file 1: Figure S2b–S2e). Adult autotetraploids have increased branch numbers, enlarged cells, larger stomata and seed sizes, lower fertility (Zhang et al. 2015; Zhang et al. 2019; Li et al. 2020), and lower growth rates in young seedlings (Dudits et al. 2016; Allario et al. 2011).

### Increasing Densities of Euchromatin Accessibility in the Autotetraploid Rice Genome

Studies in *Arabidopsis* have shown that genome doubling affects chromatin architecture; in particular, autotetraploids presented more interchromosomal interactions and fewer short-range chromatin interactions than diploid progenitors (Zhang et al. 2019). However, the intrinsic feature of the relationship between ACRs and autopolyploidization is still undetermined. Therefore, we applied ATAC-seq to aboveground tissues containing young leaves and stems of both autotetraploid rice and its diploid parent. As a result, 29,412, 37,998, and 24,886 peaks for 2 × rice and 36,135, 39,383, and 33,633 peaks for 4 × rice were called from three replicates of ATAC-seq and displayed strong Pearson correlation coefficients (R^2^ > 0.9) (Additional file 2: Table S1; Additional file 1: Figure S3a and b), suggesting that the data are reliable with high reproducibility. Subsequently, we identified 17,433 and 18,013 ACRs derived from these peaks in diploid and autotetraploid rice (Additional file 3: Table S2). Moreover, the repeatability of these ATAC-seq datasets can also be illustrated by their decoration patterns on ~ 80 kb chromatin region (Additional file 1: Figure S3f). Together, our data provide an overview of repeatable and reliable ACRs in 2 × and 4 × rice.

**Fig. 2:**
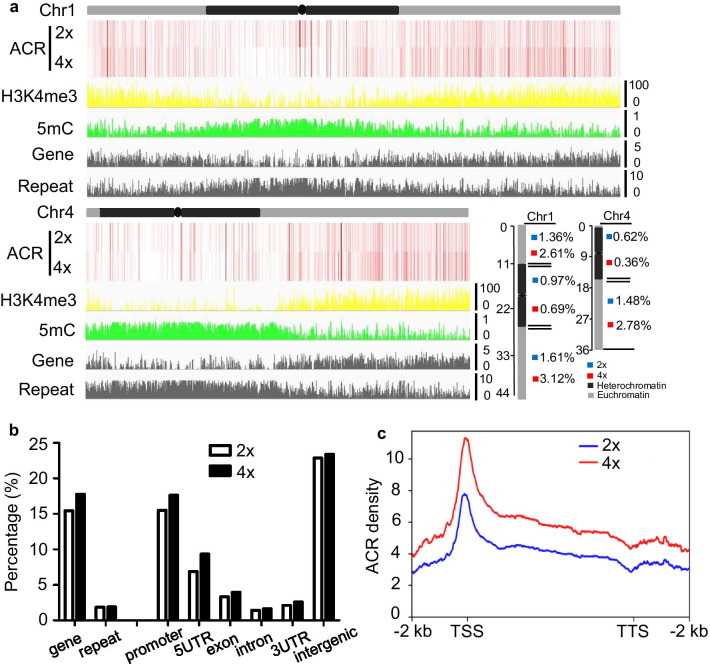
Landscape of ACRs in diploid and autotetraploid rice. **a** The distribution of ACRs in diploid (2×) and autotetraploid (4×) rice on chromosomes 1 and 4. The average densities of ACRs per 1 kb bin were calculated (*P* value < 0.05) and are shown in the vertical bars. Relative density of gene and repetitive DNA were used to defined as euchromatin and heterochromatin. As shown, the black bars represent heterochromatin and grey ones represent euchromatin. The average levels of H3K4me3 (SRR6781461) and 5mC (GSE121274) are shown for comparison. The percentages of ACR density of heterochromatic and euchromatic regions of 2 × and 4 × rice chromosomes are shown in the lower right panel. H3K4me3 is enriched in euchromatin, whereas 5mC marks heterochromatin. In this regard, the chromosomes are colored to illustrate the regions of heterochromatin (dark black) and euchromatin (light black) according to the chromosomal distributions of H3K4me3 and 5mC, respectively. **b** The proportions of ACR-related genes and repeats (histogram; left) and genomic regions (right) in 2 × and 4 × rice leaves based on ATAC-seq from three biological replications. UTR, untranslated region. **c** ACR density profile in genes. The 2-kb upstream and downstream flanks are aligned. The average ACR densities per bin, which were calculated from the Tn5 integration frequency within each 100 bp interval, are plotted. *TTS* transcription termination site

In rice chromosomes, the majority of heterochromatin is distributed in the pericentromeric regions, with chromosome 4 having a distinct pattern in which the entire left (short) arm is highly heterochromatinized (Cheng et al. 2001). We observed that ACRs tend to be enriched in euchromatic regions (Fig. 2a), consistent with our previous observations in the sorghum genome (Zhou et al. 2020). For comparison, we analyzed genome-wide 5 mC (a heterochromatic gene mark; Fig. 2a; GSE121274) and H3K4me3 (a euchromatic gene mark; Fig. 2a; SRR6781461) in the same tissues of wild-type plants. While more H3K4me3 peaks were found in euchromatic regions, 5 mC was relatively more enriched in the heterochromatic and pericentromeric regions (Fig. 2a), confirming previous data (Tan et al. 2016). The analysis revealed that the rice autopolyploidization resulted in clear gains of ACRs in euchromatic regions of the chromosomes, and to a lesser extent, slight losses in heterochromatin regions (Fig. 2a). Thus, our data indicated that genome duplication is involved in the ACRs of both heterochromatic and euchromatic regions.

In detail, we found that a higher frequency of ACRs occurred in autotetraploid rice genes (17.8%) than in diploid rice genes (15.5%), while this phenomenon was not observed in 2 × and 4 × rice repeats (Fig. 2b). To study the effects of duplication on the chromatin accessibility of different genomic elements, we calculated the percentages of ACR-associated promoters, 5′ untranslated regions (5′UTRs), 3′UTRs, coding exons, introns and intergenic regions. In general, ACRs in 2 × and 4 × rice were both located in much higher proportions in the promoter, intergenic, and 5′UTR regions than in other regions in gene bodies (Fig. 2b), which was similar to the distribution pattern of ACR in sorghum gene elements (Zhou et al. 2020). Moreover, ACRs in the autotetraploid rice promoter and 5’UTR exhibited a clear increase in contrast to diploid rice (Fig. 2b), which suggested that genome doubling may be a mechanism that promotes the expression of genes in 4 × rice in response to genome-dosage effects following WGD. To analyze the autopolyploidization effects on ACR density in different regions of rice protein-coding genes, we calculated the average ACR density for every 100-bp interval of each gene and its 2-kb upstream and downstream flanking regions in 2 × and 4 × plants. The analysis revealed that, in both 2 × and 4 × rice, ACRs were mainly present around TSSs in contrast to other genic regions (Fig. 2c), which was in line with the observation of much lower occurrences of the ACRs in the 3′UTR (Fig. 2b). Clearly, autotetraploid rice displayed a higher ACR density than diploid rice (Fig. 2c). Collectively, these data indicated that rice autopolyploidization may have a function in modulating chromatin accessibility.

### Positional ACR Genes are Enriched in Specific Biological Pathways as a Result of Autotetraploidization in Rice

The functional roles of ACRs in transcriptional regulation provide physical scaffolds to recruit transcriptional coregulators and/or chromatin remodelers (Klemm et al. 2019; Zhou et al. 2020; Lu et al. 2019). However, the mechanism by which genome duplication controlling gene expression is dependent on ACRs remains unclear. To address this issue, two sets of RNA-seq data were obtained from three replicates of the same stages of 2 × and 4 × rice (R^2^ > 0.9; Additional file 1: Figure S3c and d; Additional file 2: Tables S1 and Additional file 4: Table S3). In addition, to reevaluate the differentially expressed genes (DEGs) recognized by DEseq2 (Love et al. 2014), we introduced Cuffdiff (Trapnell et al. 2010) and EdgeR (Robinson et al. 2010), which are alternative analyses for RNA-seq. Consistently, the vast majority of DEGs (Additional file 4: Table S3) in DEseq2 overlapped with DEGs in edgeR (above 95%), but only one-third of the DEGs called in Cuffdiff overlapped with the DEGs from DEseq2 and edgeR (Additional file 1: Figure S4a and S4b). In contrast with Cuffdiff, the fold change of DEGs from DEseq2 and edgeR displayed strong Pearson correlation coefficients (R^2^ = 0.994; Additional file 1: Figure S4c). Moreover, the RNA-seq data were validated by qPCR tests of randomly selected genes (Additional file 1: Figure S4d), which revealed a high correlation with the RNA-seq results (R^2^ = 0.655; Additional file 1: Figure S4e). Thus, DEseq2 should be suitable to calculate differentially expressed genes in 2 × and 4 × rice with the classical normalization method according to previously described protocols (Zhang et al. 2019).

**Fig. 3:**
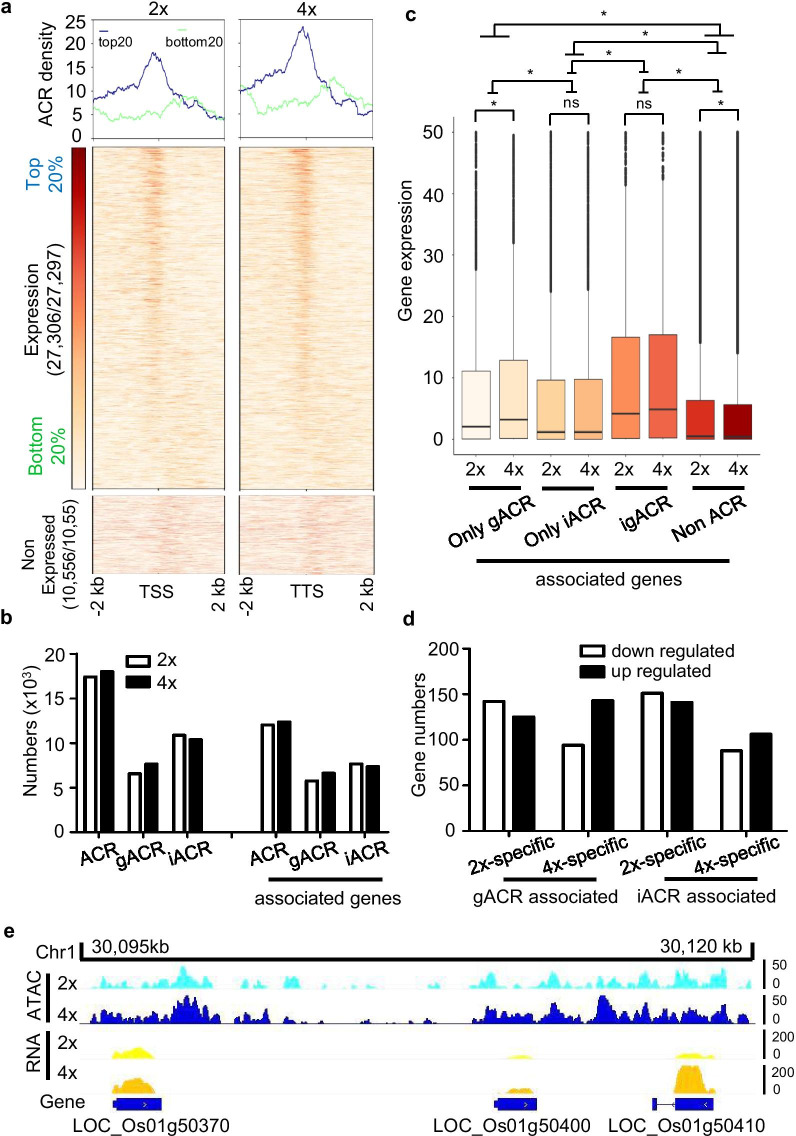
ACRs are associated with transcriptional regulation in autotetraploid rice. **a** Heatmap showing the distribution of ACR read counts from 2 × and 4 × rice that were arranged in descending order based on FPKM values. The transition from white to red corresponds to ACR read intensities from low to high levels. Expressed genes with the top 20% (blue) and bottom 20% (green) transcriptional levels are shown. **b** Numbers of leaf ACRs and its associated genes. The ACRs are defined based on their distance to the nearest gene and are categorized as genic (gACRs; overlapping at least 1b with a gene), proximal (pACRs; within 2 kb of a gene, light blue), distal (dACRs; > 2 kb from a gene, dark red), or intergenic (iACR; the sum of pACRs and dACRs, no overlapping genes). **c** Boxplots of the expression levels of different types of associated ACRs. Only gACR- and only iACR-associated genes overlapped with gACRs and iACRs, respectively; igACR-associated genes were associated with both intergenic and genic ACRs; and nonACR-associated genes (neither gACR nor iACR) did not overlap with genes. Asterisks indicate significance of differences at a *P *value < 0.05 by Wilcoxon rank sum test. The five statistical values of the boxplot from top to bottom are the maximum, third quartile, median, first quartile, and minimum. The centerline is the median, the box limits are the upper and lower quartiles, and the whiskers are the 1.5-times interquartile ranges. **d** Numbers of up-regulated and down-regulated genes associated with gACRs and iACRs, which were only detected in 2× (2×-specific) or 4× (4×-specific) rice. **e** Snapshots of ACR distributions and gene expression (Scaffold: Chr1, 30,095–30,120 kb). The ACR density and expression levels are observed and scaled by the y-axis

These data revealed that active genes that showed higher expression levels (top 20%) exhibited typical sharp peaks of ACRs at TSSs; however, no obvious peaks were observed in inactive genes (bottom 20%), which showed weak expression levels (Fig. 3a). These data indicated that the accumulation of ACRs around the TSS contributed to positive control of gene transcription in rice. Moreover, compared to the promotion of ACR in modulating transcriptional processes in diploid rice, ACR in autotetraploid rice displayed a much greater ability to positively control gene expression (Fig. 3a). Overall, these analyses indicated that gene expression in autotetraploid rice was associated with chromatin accessibility.

Next, we classified ACRs based on their proximity to the nearest annotated genes. As a consequence, 6555 (37.6%) and 7641 (42.4%) of the ACRs in 2 × and 4 × rice, respectively, were designated genic ACRs (gACRs) because they overlapped with the nearby annotated genes by at least 1 bp (Fig. 3b; Additional file 3: Table S2). In addition, 10,878 (62.4%) and 10,372 (57.5%) of the ACRs in 2 × and 4 × rice, respectively, were designated intergenic ACRs (iACRs) because they were at least 1 bp away from nearby genes (Fig. 3b; Additional file 3: Table S2). Accordingly, there was a noticeable increase in gACR-associated genes in 4 × rice (6603) and no obvious change in either ACR- or iACR-associated genes compared to the total number of genes in 2 × rice (5751) (Fig. 3b; Additional file 5: Table S4). Next, we characterized the basic genomic features of the various types of ACRs. In general, the sizes of full-length 4 × rice ACR transcripts were shorter than those of 2 × rice transcripts (Additional file 1: Figure S5a). The results showed no obvious differences in A/T-rich regions in autotetraploid rice and its diploid parent (Additional file 1: Figure S5b). In retrospect, these distinct discrepant traits of ACRs were found in 2 × and 4 × rice, indicating that ACRs appear to have a distinct function during rice autopolyploidization.

To better understand the correlations of gene expression levels with different types of ACRs in 2 × and 4 × rice, we classified all genes into four categories: (1) genes associated with only gACRs (only gACRs), (2) genes associated with only iACRs (only iACRs), (3) genes associated with gACRs and iACRs (igACRs), and (4) genes without ACRs (nonACRs). Then, we calculated the transcriptional levels of these groups of genes, and the results showed that the average expression levels of genes with only gACRs or only iACRs were significantly higher than those of genes without ACRs (Fig. 3c). In fact, we observed that only gACR-associated genes displayed significantly lower expression levels than igACRs but higher expression levels than only iACRs (Fig. 3c). These results were consistent with previous observations in sorghum (Zhou et al. 2020). The analysis revealed that genic ACRs play predominant roles in controlling gene transcription in the rice genome. Additionally, only gACR-associated genes in 4 × rice displayed higher transcriptional activity than those in 2 × rice, which was not observed in other groups of genes (Fig. 3c). To further refine the correlation of the DEGs and differential ACR-associated genes (DAGs), we defined gACR-associated genes that specifically existed in 2× (2×-specific) or 4× (4×-specific) plants, similar to iACRs (Additional file 1: Figure S6a). In terms of DEGs (Additional file 1: Figure S6b), including 1330 upregulated genes and 1317 downregulated genes, autotetraploid rice had nearly 150 upregulated 4×-specific gACR-associated genes, which was more than the number of downregulated genes (Fig. 3d). In contrast, obvious differences were not found in other overlapping genes between DAGs and DEGs (Fig. 3d). As an example, the expression and ACR distributions of representative transcriptionally normalized reads corresponding to gACRs, iACRs, and igACRs in 2 × and 4 × rice are illustrated (Fig. 3e). In total, the analysis showed that transcriptional regulation of ACRs was also associated with their own positional states in the rice genome. In contrast with intergenic ACRs, genic ACRs were able to modulate transcriptional activity, which was much more prominent during rice genome duplication.

Consistently, Gowinda analysis revealed that genes involved in DNA-binding transcription factor activity, regulation of gene expression, response to biotic stimulus and membrane part were enriched (*P* value < 0.05) for gACRs that specifically exist in 4 × rice (Additional file 6: Table S5). These processes were also enriched for gACR-associated genes in autotetraploids, whereas none of the genes associated with any GO terms were enriched for those in 2 × rice (Additional file 6: Table S5). In particular, these differentially expressed genes are associated with responses to stimuli in autopolyploid plants (Zhang et al. 2019; Allario et al. 2011). Thus, these analyses reinforced the hypothesis that gACRs were able to promote transcription, which was enhanced in the process of rice genome doubling.

### The Involvement of ACRs in Transcriptional Regulation is Associated with H3K36me2 and H3K36me3 During Rice Autopolyploidy

**Fig. 4:**
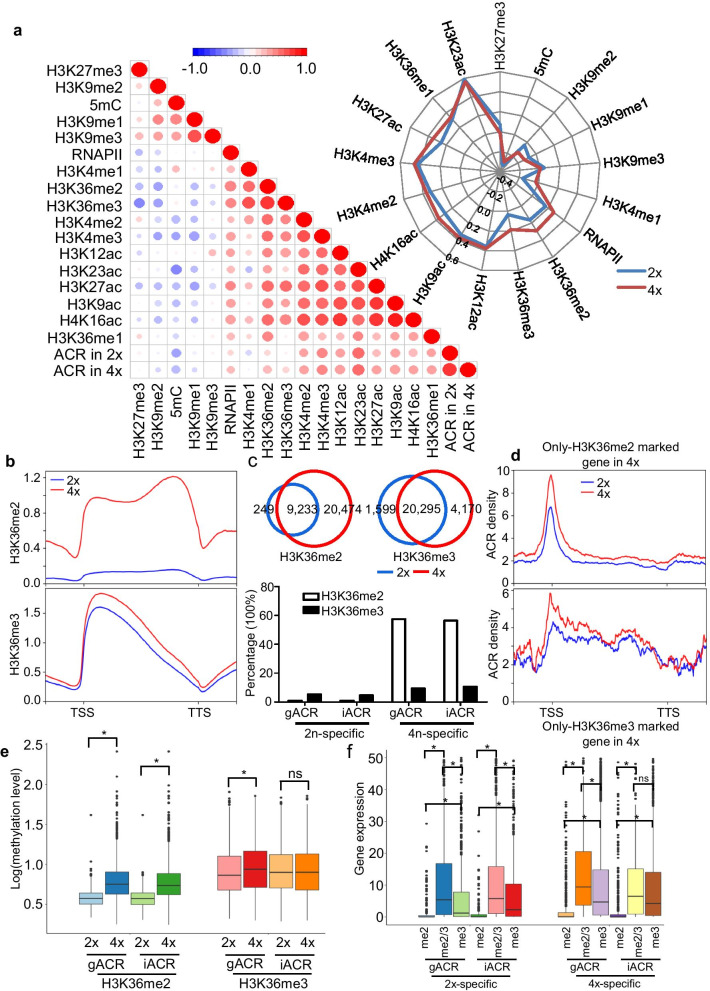
Comparison of specific ACR distributions detected in diploids and autotetraploids with other epigenomic marks in the rice genome. **a** Correlogram of ACRs in 2 × and 4 × and other epi-marks with transcriptional regions. The Spearman coefficient is indicated by the color and size of each point. Red represents high correlation, and blue represents low correlation. In detail, the radar map shows the positive correlations of ACR with active epigenetic marks and the negative correlations with silent marks. **b** Average genome-wide occupancies of H3K36me2 (top row) and H3K36me3 (bottom row) in 2 × and 4 × rice leaves. The 2-kb upstream and downstream flanks are aligned. **c** Venn diagram of H3K36me2- and H3K36me3-marked genes in diploid and autotetraploid rice (top row) and percentages of the two marks associated with the four types of genes defined in Fig. 3d. **d** Profile of ACR density within only H3K36me2 (top row) and H3K36me3 (bottom row)-marked genes in 2 × and 4 × rice. **e**, **f** Box plots of the methylation levels (**e**) and transcriptional level (**f**) of genes marked by H3K36me2 and H3K36me3. Asterisks indicate significant differences at a *P* value < 0.05 by Wilcoxon rank sum test. The five statistical values of the boxplot from top to bottom are the maximum, third quartile, median, first quartile, and minimum. The centerline is the median, the box limits are the upper and lower quartiles, and the whiskers are the 1.5-times interquartile ranges. *ns* not significant

The interrelationship between histone marks with ACRs has been studied in *Arabidopsis* and other plants, suggesting that the interplay of ACRs and histone marks is worth studying in plants (Lu et al. 2019; Sun et al. 2019; Frerichs et al. 2019; Zhou et al. 2020). However, the crosstalk between ACRs and histone modification during plant genome autopolyploidization is still unclear. To investigate the epigenomic signatures of chromatin in relation to ACRs in the rice diploid and autotetraploid genomes, we integrated our ATAC-seq data in 2 × and 4 × rice with ChIP-seq and MeDIP-seq data (Additional file 7: Table S6). Pearson correlation coefficients were also introduced to indicate the degree of concurrence of histone marks at transcript regions, which demonstrated that ACRs in either 2 × or 4 × rice preferred to be associated with active marks, other than the well-known inactive and heterochromatic modifications H3K9me2, H3K27me3, and 5mC (Fig. 4a). These data were in accordance with previous observations that ACRs contributed to the positive control of gene transcription in plants (Zhou et al. 2020; Lu et al. 2019). More interestingly, the correlogram suggested that ACRs in 4 × rice preferred to recruit the H3K36me2 and H3K36me3 marks due to their positive correlations with ACRs (Fig. 4a). Similarly, the correlations of ACRs and epi-marks in the whole doubled genome appeared to be similar to those in transcriptional regions to some extent (Fig. 4a and Additional file 1: Figure S7). Collectively, the analysis supported that H3K36me2/3 act as predominant chromatin marks during rice genome autopolyploidization.

To analyze the effect of genome duplication on histone methylation levels at different regions of rice protein-coding genes, whole-genome profiles of two histone marks (H3K36me2 and H3K36me3) from two replicates of ChIP-seq in 2 × and 4 × rice that displayed strong Pearson correlation coefficients (R^2^ > 0.8; Additional file 1: Figure S3e and S3f) were examined, while the non-ChIP genomic DNA was included as input. As a result, a large number of histone modification peaks (from 10,836 to 92,633) were called (Additional file 2: Table S1). We observed that the evolutionarily conserved active mark H3K36me3 was abundant near the transcriptional start site (Fig. 4b); in contrast, H3K36me2 was distributed evenly across the transcriptional region (Fig. 4b), indicating that these two types of H3K36me marks may have different roles. Interestingly, the augmented levels of H3K36me2 and H3K36me3 were associated with rice autopolyploidization, especially H3K36me2 (Fig. 4b), which was in line with the observation of a correlation between multiple epi-marks and ACRs (Fig. 4a). In detail, 20,474 and 4,170 genes, respectively, were found to be only marked with H3K36me2 and H3K36me3 in 4 × rice (Fig. 4c). In addition, compared to 2 × rice, our findings showed that a dominant proportion (no less than 50%) of H3K36me2-marked genes were found for gACR- or iACR-specific genes in 4 × rice (Fig. 4c). At the same time, the percentages of H3K36me3-marked genes that were associated with gACRs or iACRs were also slightly increased in 4 × rice (Fig. 4c). Unsurprisingly, the ACR density levels of only H3K36me2- and H3K36me3-associated genes in autotetraploid rice were higher than those in diploid rice (Fig. 4d). Additionally, the enrichment of H3K36me2 in either gACR- or iACR-associated genes, which were specific to 4 × rice, was higher than that in 2 × rice; in contrast, H3K36me3 appeared to be slightly ameliorated in gACR-specific genes in 4 × rice compared to 2 × rice (Fig. 4e). These results indicated that methylation of both H3K36me (H3K36me2/3) was required for rice genome duplication and that H3K36me2 may play a dominant role in functional regulation.

Then, we examined how and to what extent ACRs and H3K36me2/3 modulate transcriptional activity in 2 × and 4 × rice. Consequently, three chromatin states were generated from classified genes that were associated with gACRs or iACR in 2 × rice and 4 × rice: only H3K36me2 (me2), combination of H3K36me2 and H3K36me3 (me2/3), and only H3K36me3 (me3). Overall, for gACR-related or iACR-related genes, me2/3-marked genes displayed significantly higher expression levels (Fig. 4f; *P* value < 0.01 by Wilcoxon rank sum). On average, me3 genes were correlated with higher gene activity in 2 × and 4 × rice, respectively, compared with me2 genes, suggesting that the combining effect (coenrichment) of H3K36me2 and H3K36me3 ought to be correlated with higher transcriptional activity (Fig. 4f; *P* value < 0.01 by Wilcoxon rank sum). Interestingly, compared with that of genes covered with 2×-specific genic ACRs, the transcriptional level was obviously enhanced in 4×-specific gACRs associated with H3K36me2 and H3K36me3 co-marked genes or only H3K36me3-marked genes (Fig. 4f), suggesting that doubling the genome induced H3K36me2 and H3K36me3 marks that were related with the higher transcriptional activity. However, the expression of only H3K36me3-marked 4x-specific iACR-associated genes showed no significant differences from H3K36me2/3-marked genes and 2×-specific iACR genes (Fig. 4f). These data indicated that, in contrast with intergenic ACRs, genic ACRs play dominant roles in the regulation of gene expression during rice doubling based on the two types of H3K36 histone methylation. Taken together, these analyses suggested that within transcriptional regions, the combination of H3K36me2 and H3K36me3 may be associated with chromatin accessibility in rice autopolyploidization.

### Rice Genome Duplication Modulates Global Metabolic Profiling

Autopolyploidization can influence metabolism in some plants (Tan et al. 2019). In rice, however, the effect of autotetraploidization on the accumulation of these metabolites has rarely been investigated. To determine whether genome doubling has an impact on rice metabolism, we sampled aerial tissues at the same stage and performed unbiased global metabolic profiling based on HPLC-Q-TOF/MS (Additional file 1: Figure S8a). A total of 119 metabolites that matched known biochemical parameters were detected (Additional file 8: Tables S7). The replicates of metabolic data showed strong Pearson correlation coefficients (R^2^ > 0.85) (Additional file 9: Figure S8b), suggesting that the data outputs were reliable with high reproducibility.

**Fig. 5:**
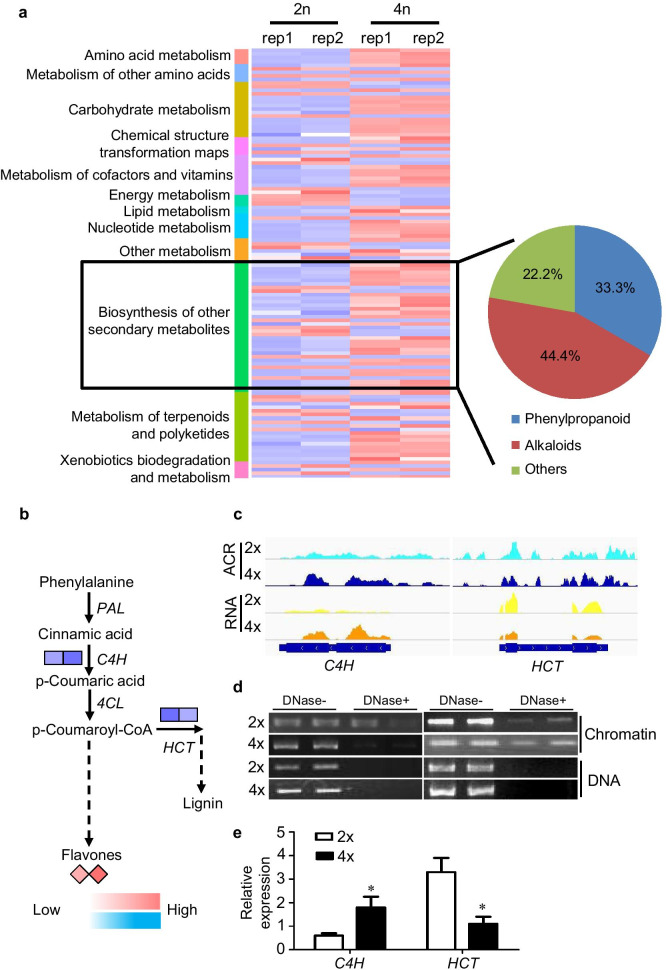
The effect of differentially expressed ACR-associated genes on metabolites in diploid and autotetraploid rice. **a** Heatmap representation of autotetraploid-induced differentially accumulated metabolites (DAMs). The level of a given metabolite was judged to increase or decrease (*P* value < 0.05) by comparing the scaled amount in autotetraploid rice with that of its diploid parent. Metabolites are categorized according to compound classes. The percentages of compounds in secondary metabolites are shown on the right. **b** Diagrammatic representation of transcriptional and metabolic levels of genes involved in phenylpropanoid metabolism in diploid and autotetraploid rice. Blue represents gene expression, and red represents metabolites. **c**–**e** A combined analysis of ACRs and gene expression of *C4H* and *HCT* in 2 × and 4 × rice. ACR and gene expression data for the *C4H* and *HCT* genes are shown for 2 × and 4 × rice. The scales are shown (**c**). DNase I-PCR shows differential DNase I sensitivity of chromatin in selected genomic regions. Equal amounts of chromatin and purified DNA were subjected to gradient DNase I treatments at final concentrations of 0 (DNase−) and 0.5 (DNase+) units mL-1. Extracted DNA was used as a template for PCR for one ACR with two replicates (**d**). qPCR validation of *C4H* and *HCT* expression in diploid and autotetraploid rice was normalized using the signal from the actin gene. The average ± SD values from three biological repeats are shown, *P value < 0.05, two-sided *t* test (**e**)

To develop a systematic approach based on global metabolomics profiling, different metabolic patterns were identified for 2 × and 4 × rice leaves. During rice genome doubling, obvious changes (more than twofold; *P* value < 0.05) were detected for specific compounds (Fig. 5a), including 83 upregulated and 36 downregulated DAMs (differential accumulated metabolites; Additional file 1: Figure S9; Additional file 8: Tables S7). Obviously, increased metabolites in various pathways were induced during rice genome duplication (*P* value < 0.05). Among these accumulations in 4 × rice, the number of secondary metabolites was the highest (Additional file 1: Figure S9). Intriguingly, secondary metabolites (also called “specialized metabolites”) play roles in core plant processes (Yuan and Grotewold 2020), including phenylpropanoids and alkaloid synthesis (Dong and Lin 2021). In detail, more than 75% of the differentially accumulated secondary metabolites were related to phenylpropanoid and alkaloids (Fig. 5a). It was previously observed that flavonoid contents were increased in autopolyploid Hylocereus (Fig. 5a). It was previously observed that contents of flavonoids were increased in autopolyploid *Hylocereus* line (Cohen et al. 2013), and upregulated genes were associated with secondary metabolism in *Stevia rebaudiana* autotetraploids compared to diploids (Xiang et al. 2019). One-third of the identified secondary metabolites were phenylpropanoids (Fig. 5a), which are of vital importance for plant development and survival (Dong and Lin 2021). Among the multiple regulatory mechanisms of phenylpropanoid metabolism, transcriptional regulation plays a central role in the regulation of the biosynthesis of phenylpropanoid metabolites and explains almost all the regulatory effects (Yuan and Grotewold 2020; Dong and Lin 2021). To monitor the ACR density and transcriptional levels in diploid and autotetraploid rice leaves, we first mapped DEGs and DAM in the rice phenylpropanoid pathway (Fig. 5b). We observed that cinnamic acid 4-hydroxylase (C4H), which is a cytochrome P450-dependent monooxygenase, and hydroxycinnamoyl transferase (HCT), which leads to the biosynthesis of two major lignin building units, displayed significantly differential transcriptional levels (Fig. 5b). As a result, flavones were detected at much higher levels in autotetraploid rice leaves than in diploid rice leaves (Fig. 5b). Moreover, as shown by the Genome Browser (Fig. 5c), our ATAC-seq and RNA-seq data suggested that the levels of ACR density and expression of C4H were higher in 4 × rice than in 2 × rice; in contrast, the ACR density of the HCT gene and its transcriptional level were higher in diploid rice. Accordingly, DNase-digested PCR and quantitative real-time PCR results confirmed these results (Fig. 5d, e). Together, a positive connection was detected between these metabolites and rice autopolyploidization, suggesting that metabolite accumulation could be used as a biomarker for plant genome duplication and providing some insights into the phenotypic changes induced by rice genome doubling.

## Discussion

To satisfy the requirements of future food security, the demand for food will increase by 50% by 2050, which will put tremendous pressure on increasing the yield per unit area (Bailey-Serres et al. 2019). Polyploidy generally originates from whole-genome duplications or interspecific hybridizations, which serve as a prevalent trend in the evolution in flowering plants. Polyploid plants often have significant advantages in terms of biomass, vigorousness, and robust adaptation to environmental changes (Van de Peer et al. 2017). Thus, crop polyploidization may play an important role in next-generation crop improvement aimed at facing food security challenges (Yu et al. 2021). Unlike allopolyploids, which can be confounded by the entanglement of whole genome duplication and hybridization, autopolyploids are created via WGD, ruling out disturbances from incompatible genomes (Zhang et al. 2015). In this regard, autopolyploid species, which carry multiple similar chromosome sets, provide an extraordinary opportunity to provide insights into genomic changes that occur in response to ploidy levels.

To better characterize WGD-induced variation in chromatic accessibility during recent autopolyploid formation in flowering plants, we performed highly integrated maps of chromatin accessible regions and the transcriptome in autotetraploid rice and its diploid donor. We obtained a comprehensive view of abundant genomic changes in chromatin accessible regions, gene expression and metabolite responses to subsequent genome duplication. Our findings indicated that genome doubling can induce genome-wide diversification of chromatin accessibility and revealed that such changes may be related to transcriptional activity and further affect metabolites, indicating that it is an effective pathway to overcome genome-dosage effects produced by autopolyploidization.

Chromatin patterning after allopolyploidization is well established (Wendel et al. 2018), but relatively little is known about the effects of autopolyploidization. Ploidy-induced chromatin accessibility changes have been reported in *Arabidopsis* (Karaaslan et al. 2020) and wheat (Lu et al. 2020). Similarly, during *Arabidopsis* somatic polyploidization, dark- and light-treated samples in 2C nuclei do not exhibit any different chromatin accessibility landscapes, whereas changes in 16C can be linked to transcriptional changes involved in the light response (Karaaslan et al. 2020). In the allopolyploid wheat genome, chromatin of the smaller D genome is more accessible than that of the larger A and B genomes. Chromatin states of different TEs vary among families and are influenced by the TEs’ chromosomal position and proximity to genes (Jordan et al. 2020). In addition, chromosome-wide patterns of reduced chromatin accessibility of genes in the hexaploid wheat chromosome arm compared with its diploid progenitor were correlated with both reduced gene expression and the imposition of new patterns of gene expression (Lu et al. 2020). Given that allopolyploids were affected by both hybridization and polyploidization, these analyses did not accurately reflect the intrinsic nature of transcriptional regulation induced by ploidy. In this regard, to study the impact of genome doubling without interspecies hybridization effects, we used autotetraploid rice induced from a diploid cultivar and independently self-pollinated over 10 generations to overcome the challenges of allopolyploid rice. The autotetraploid rice exhibited remarkable differences in morphological traits compared with diploid rice and decreased leaf, root and whole plant sizes in young seedlings (Fig. 1a), even though the midvein was significantly thicker (Fig. 1b) and the cortex cell area was greater (Fig. 1c) in 4 × rice. At mature stages, autotetraploid rice displayed increased plant height and panicle and grain sizes (Additional file 1: Figure S2), which are similar to other polyploidy plants (Zhang et al. 2015, 2019; Li et al. 2020; Dudits et al. 2016; Allario et al. 2011). Similarly, autotetraploid rice from another *indica* diploid cultivar displayed similar phenotypes at mature stages, including increased leaf sizes, decreased fertility, fewer branches, reduced spikelet numbers, and enlarged grain sizes (Zhang et al. 2015). Moreover, despite strong biomass production and higher resistance against abiotic and biotic stresses (Wu et al. 2013; Wang et al. 2021), autotetraploid rice has poor seed set, which has become the largest bottleneck and the major barrier in commercial production (Wu et al. 2014; Guo et al. 2017). Partial pollen and embryo sac sterilities are the two important factors that lead to low seed set in autotetraploid rice (Li et al. 2017; Wu et al. 2014). Because excellent salt tolerance is enhanced in tetraploid rice (Wang et al. 2021), we also noticed that the genes related to physiological and ecological tolerance were changed in autotetraploids, which might be related to the environmentally adaptive phenotypes of autotetraploids (Additional file 6: Table S5).

In the present study, we began with ATAC-seq to characterize differential distribution patterns of ACRs in autotetraploid rice compared to its diploid donor, coupled with genome-wide transcriptional profiling as well as global metabolic data. Our data revealed that ACRs obviously accumulated in euchromatic regions of the doubled rice chromosomes and, to a lesser extent, were slightly lacking in heterochromatin regions (Fig. 2a). In line with previous results showing that ACRs are mostly enriched in euchromatin and promote transcription (Lu et al. 2019; Zhou et al. 2020; Ricci et al. 2019), WGD-induced chromatic accessibility may be associated with gene expression, which may occur in euchromatin. It was also verified that a higher frequency of ACRs occurred in autotetraploid rice genes, which was not observed in 2 × and 4 × rice repeat regions (Fig. 2b). On average, autotetraploid rice displayed a higher ACR density than diploid rice (Fig. 2c), implying that genome doubling may be involved in controlling chromatin accessibility along transcriptional regions.

As a prevalent hallmark of regulatory DNA in flowering plants (Lu et al. 2019), the accumulation of ACRs at the TSS of a gene contributed to the positive control of gene transcription in sorghum, whereas correlations were not observed for genes whose ACRs were located at the TTS (Zhou et al. 2020). Consequently, in comparison with the functional regulation of ACRs in diploid transcription, WGD-induced ACRs displayed a much greater ability to positively control gene expression (Fig. 3a). Thus, these analyses indicated that gene expression in autotetraploid rice was associated with chromatin accessibility. In addition, given that the positional effects of ACRs contributed to gene activity in plants (Lu et al. 2019; Zhou et al. 2020), we observed that only gACR-associated genes in 4 × rice displayed higher transcriptional activity than those in 2 × rice, which was not observed in other types of ACR-associated genes (Fig. 3c). The number of upregulated genes which were 4×-specific gACR-associated genes was higher than that of downregulated genes (Fig. 3d). Genic ACRs preferred to modulate transcriptional activity, which was much more prominent during rice autopolyploidization. The polyploidy genome is dynamic and undergoes structural alterations (Otto 2007). Recently, in *Arabidopsis*, autotetraploid has been shown to have a relatively lower compactness in chromosome arms than its diploid progenitor (Zhang et al. 2019). In addition, approximately 16% of topologically associated domains in each diploid are reorganized in the respective tetraploid subgenomes, which affects the transcriptional activity of abundant genes (Wang et al. 2018). Therefore, it is assumed that genome dosage resulted in dynamic gACRs in the process of rice genome doubling, which enhanced the functional promotion of transcription.

Epigenetic changes may provide an effective and flexible means for a polyploidy cell to overcome “genomic shock” (Chen 2007). Continuous efforts have focused on studying epigenetic variation in plant allopolyploids (Parisod et al. 2009; Ng et al. 2011; Martienssen 2010). In autotetraploid rice, an increased cytosine methylation level of DNA TEs may inactivate the transcription of neighboring genes, leading to a lack of significant differences in transcriptome alterations for most genes compared to their diploid donor (Zhang et al. 2015). More than 80% of the differentially expressed 24-nt TE-siRNAs exhibited down-regulation during the pollen development of autotetraploid rice; these expression changes may activate TEs and induce genome destabilization (Li et al. 2017). Among the DEGs, 10 genes were annotated as methyltransferases or hydroxymethyltransferases, which were related to epigenetic marks (Guo et al. 2017). These findings suggest that the irregular TEs associated with the hypo-methylation and downregulation of 24-nt TE-siRNAs result in an autotetraploid rice incompatibility response to “genomic shock” by polyploidization and probably disturb chromatin structure during autotetraploid rice meiosis. In addition, salt tolerance is enhanced in tetraploid rice through lower sodium uptake and correlates with DNA methylation controlling jasmonic acid-related genes (Wang et al. 2021). In addition to DNA methylation, genome duplication contributes to the switching of some loose and compact structural domains with an altered histone modification status, including H3K4me3 and H3K27me3 in the *Arabidopsis* genome, which modulates genome-wide transcription (Zhang et al. 2019). Similar to these results, our data further confirmed that ACRs in autotetraploid rice preferred to recruit H3K36me2 and H3K36me3 marks due to their positive correlations with ACRs, in contrast to other epi-marks (Fig. 4a and Additional file 1: Figure S7). In addition, ChIP-seq results revealed that H3K36me2 and H3K36me3 were required for rice genome doubling and that H3K36me2 may act as the predominant rules in functional regulation (Fig. 4b–e). In contrast with iACRs, gACRs display excellent correlation with H3K36me2/3 in controlling the transcriptional level during rice doubling (Fig. 4f). These results showed that the combination of H3K36me2 and H3K36me3 may be associated with WGD-induced chromatin accessibility when transcribed.

Additionally, autopolyploidization can influence metabolism in some plants (Tan et al. 2019). We conducted unbiased global metabolic profiling based on HPLC-Q-TOF/MS (Additional file 1: Figure S8a), which detected 119 well-annotated metabolites (Additional file 8: Tables S7). Among these accumulated metabolites, secondary metabolites and amino acid carbohydrate metabolism were increased in autotetraploid rice relative to diploid rice, implying that metabolite accumulation could be used as a biomarker for plant genome duplication and provide some insights into the phenotypic changes induced by rice genome doubling (Fig. 5a; Additional file 1: Figure S8).

In conclusion, we investigated dynamic ACRs following chromosome doubling in autotetraploid rice. Our study sheds light on the characterization of ACRs in autotetraploid rice compared to its diploid donor, unveils their interplay with multiple epigenetic markers, and suggests their positive roles in the regulation of transcriptional gene expression and the production of metabolites in autotetraploid plants.

## Conclusions

Collectively, the ATAC-seq results show that the effect of ACRs on transcriptional gene expression relies on their positions in the autotetraploid rice genome. In addition, integrated analysis with ChIP-seq and RNA-seq suggests that the combination of H3K36me2 and H3K36me3 may be associated with dynamic perturbation of ACRs introduced by genome doubling. Consequently, we found that numerous metabolites were stimulated by rice genome doubling. Our findings provide new insights into the modulation of chromatin accessibility for transcription during autopolyploidization, resulting in variations in rice morphology and products.

## Supplementary Information

**Additional file 1**. **Figure S1.** Flow cytometric DNA histograms for diploid (2x) and autotetraploid rice (4x). **Figure S2.** Morphological differences between diploid and autotetraploid rice at the young and mature stages. **Figure S3.** Data reproducibility in this study. **Figure S4.** Comparisons of DEGs calculated with different software. **Figure S5.** Comparisons of properties of ACRs in wild-type and autotetraploid rice leaves. **Figure S6.** Functional analysis of DAGs associated with specific gACRs and iACRs in diploid and autotetraploid rice. **Figure S7.** Correlogram of ACRs in 2x and 4x rice, as well as other epi-marks. **Figure S8.** Quality analysis of metabolite replicates. **Figure S9.** Numbers of DAMs in diploid and autotetraploid rice.

**Additional file 2**. **Table S1.** Sequenced data alignment summary.

**Additional file 3**. **Table S2.** Different types of ACRs in diploid and autotetraploid rice.

**Additional file 4**. **Table S3.** The detailed information of differential expression genes in 2x and 4x rice.

**Additional file 5**. **Table S4.** ACRs associated genes in diploid and autotetraploid rice genome.

**Additional file 6**. **Table S5.** List of KEGG and Gowinda terms of gACR-specific associated genes and up-regulated genes in diploid and autotetraploid rice.

**Additional file 7**. **Table S6.** The sequence read archive published data used in this study.

**Additional file 8**. **Table S7.** List of differential accumulated metabolites in diploid and autotetraploid rice.

**Additional file 9**. **Table S8.** Primers in this study.

## Data Availability

The raw sequence data reported in this paper have been deposited in the Genome Sequence Archive in BIG Data Center, Beijing Institute of Genomics (BIG), Chinese Academy of Sciences, under accession numbers CRA004011 and CRA004369 that are publicly accessible at https://bigd.big.ac.cn/gsa.

## Abbreviations

ATAC-seq: Transposase-accessible chromatin sequencing
ACRs: Accessible chromatin regions
ChIP-seq: Chromatin immunoprecipitation sequencing
H3K9ac: H3 lysine 9 acetylation
H3K27ac: H3 lysine 27 acetylation
H3K4me1: H3 lysine 4 monomethylation
H3K36me2: H3 lysine 36 dimethylation
H3K36me3: H3 lysine 36 trimethylation
H3K9me2: H3 lysine 9 dimethylation
H3K27me3: H3 lysine 27 trimethylation
WGD: Whole-genome duplication
PcG: Polycomb group
DAGs: Differential ACR associated genes
DEGs: Differential expression genes
GO: Gene ontology
C4H: 4-Hydroxylase
HCT: Hydroxycinnamoyl transferase
