# Supplementary material for: A Genome Doubling Event Reshapes Rice Morphology and Products by Modulating Chromatin Signatures and Gene Expression Profiling
Source: Rice (N Y). 2021 Aug 4;14:72. doi: 10.1186/s12284-021-00515-7 (PMC8339180; doi:10.1186/s12284-021-00515-7)
Supplement: Supplementary file 1 — Additional file 1. Figure S1. Flow cytometric DNA histograms for diploid (2x) and autotetraploid rice (4x). Figure S2. Morphological differences between diploid and autotetraploid rice at the young and mature stages. Figure S3. Data reproducibility in this study. Figure S4. Comparisons of DEGs calculated with different software. Figure S5. Comparisons of properties of ACRs in wild-type and autotetraploid rice leaves. Figure S6. Functional analysis of DAGs associated with specific gACRs and iACRs in diploid and autotetraploid rice. Figure S7. Correlogram of ACRs in 2x and 4x rice, as well as other epi-marks. Figure S8. Quality analysis of metabolite replicates. Figure S9. Numbers of DAMs in diploid and autotetraploid rice. [file 12284_2021_515_MOESM1_ESM.pdf]

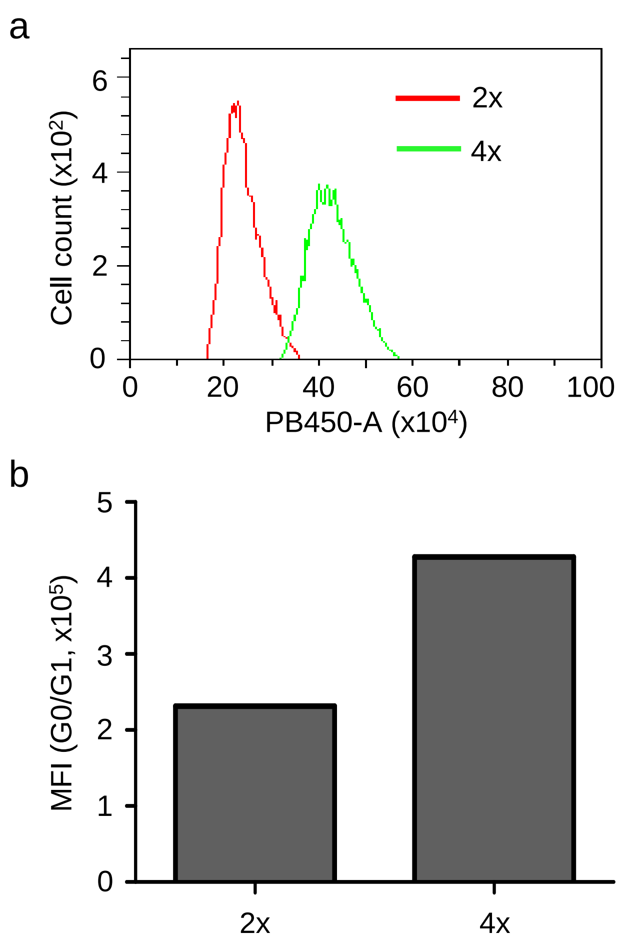

Figure S1. Flow cytometric DNA histograms for diploid (2x) and autotetraploid rice (4x).
(**a**) DNA histogram of wild-type and autotetraploid rice. The nuclei were stained with DAPI. **(b**) The MFI (median fluorescence intensity) values are shown.

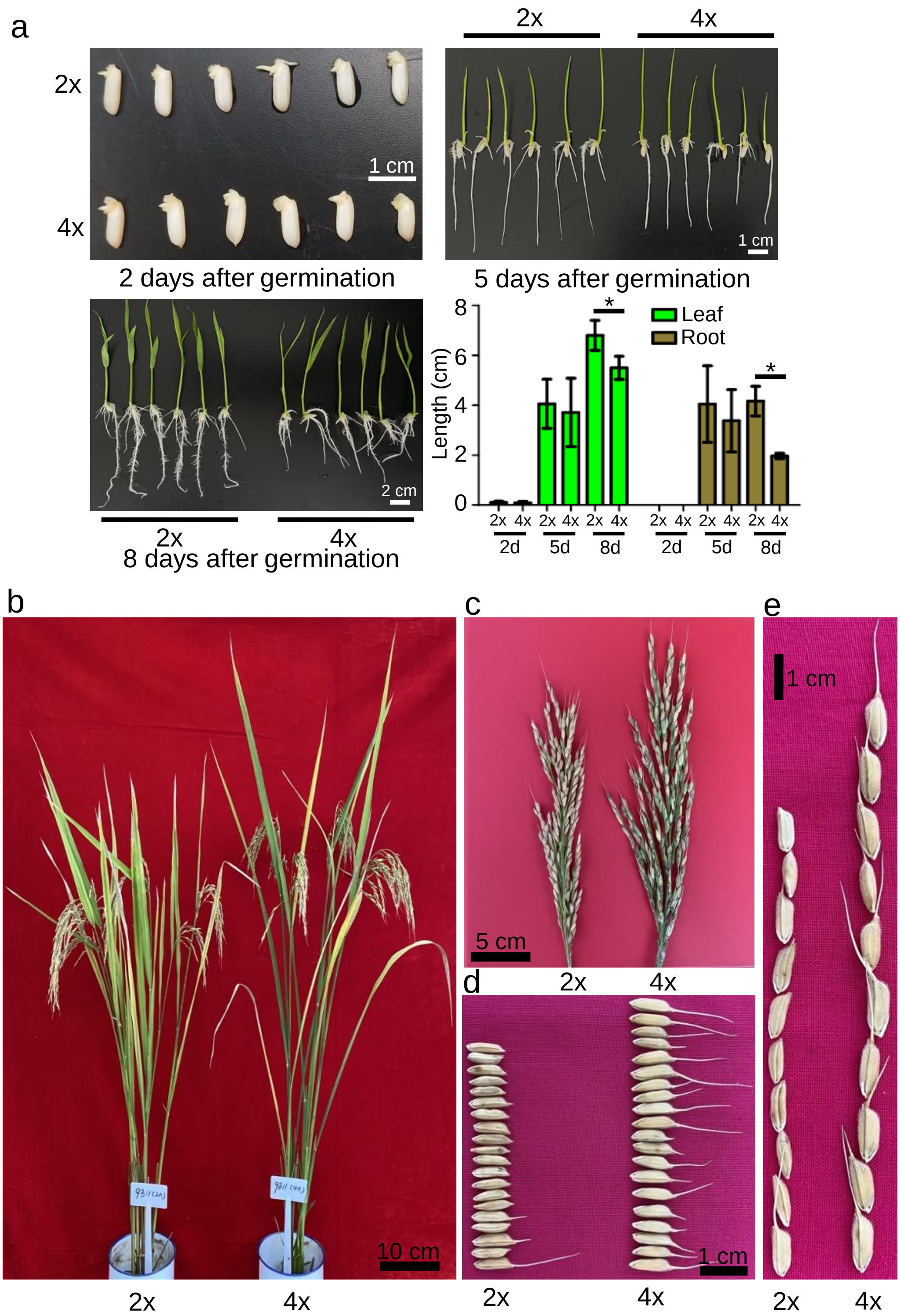

Figure S2. Morphological differences between diploid and autotetraploid rice at the young and mature stages. (a) Dynamic growth of young 2x and 4x rice seedlings at 2 days (2d), 5 days (5d) and 8 days (8d) after seed germination (scale bar, 1 cm and 2 cm). Leaf (green) and root (yellow) length measures are shown on the right. *P-value < 0.05, *t-test*, two-sided, Error bar represents SD (N = 30). (**b**) Diploid and autotetraploid plants at mature stages are shown (Scale bar, 10 cm). (**c**) Panicle morphology of diploid and autotetraploid rice (Scale bar, 5 cm). (**d and e**) Grain morphology of diploid and autotetraploid rice (Scale bar, 1 cm).

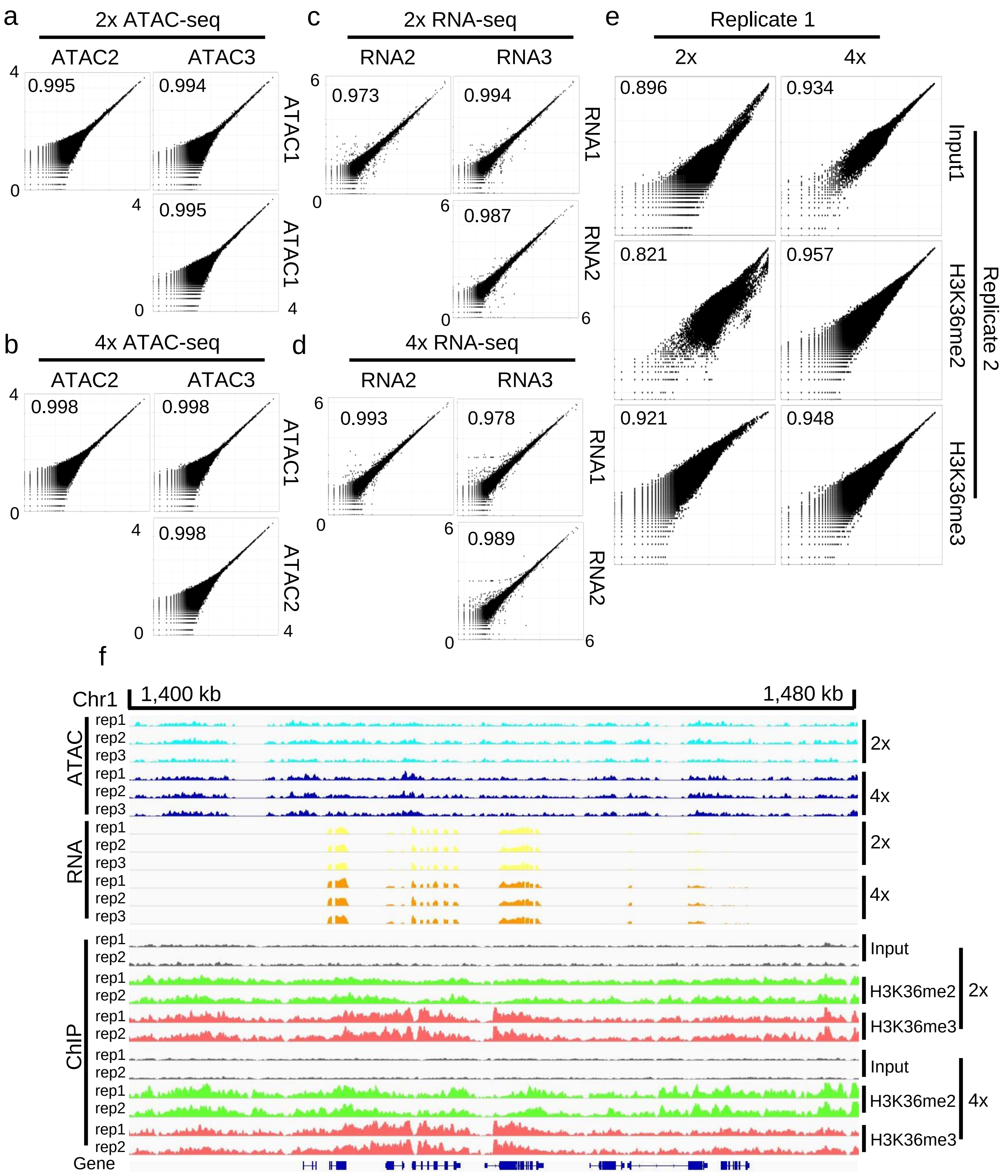

Figure S3. Data reproducibility in this study.

(**a - e**) Scatterplots of enrichment scores compared across 2x and 4x rice biological triplicates of ATAC-seq. ATAC1, ATAC2 and ATAC3 (a and b); RNA-seq, RNA1, RNA2, and RNA3 (c and d); and ChIP-seq with two replicates of Input, H3K36me2, and H3K36me3 (**e**). Pearson correlation coefficient values are shown at the top left corner of each plot. (**f**) Snapshots illustrating the enrichment of ATAC-seq (ATAC), RNA-seq (RNA), and ChIP-seq (Input, H3K36me2 and H3K36me3). The representative region is on chromosome 1 (Chr 1; scaffold: 1,400 kb - 1,480 kb).

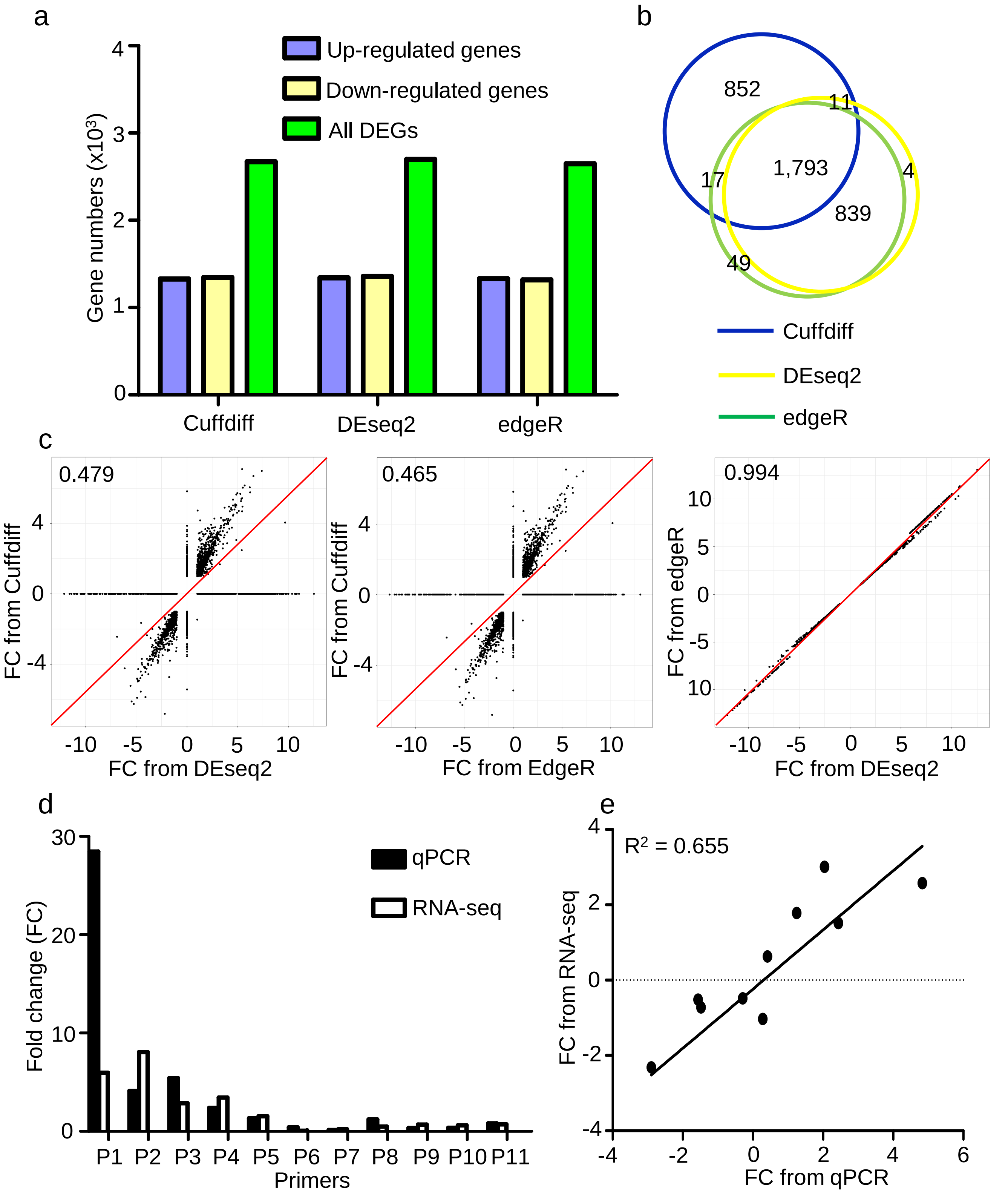

Figure S4. Comparisons of DEGs calculated with different software.
(**a**) Numbers of DEGs from three software programs, Cuffdiff, DEseq2 and edgeR (FC > 2, *P*-value < 0.05). (**b**) Overlap of DEGs calculated from these software programs. (**c**) Correlation of fold-change (FC) values of DEGs calculated with Cuffdiff, DEseq2 and EdgeR. Pearson correlation coefficient values are shown at the top left corner of each plot. (**d**) Quantitative RT-PCR of the relative transcript levels of these genes compared to actin and correlation of qPCR and RNA-seq data. Tested loci: P1: LOC_Os02g45450; P2: LOC_Os03g56160; P3: LOC_Os10g30450; P4: LOC_Os02g17534; P5: LOC_Os02g28720; P6: LOC_Os09g34250; P7: LOC_Os08g10290; P8: LOC_Os03g08600; P9: LOC_Os04g54300; P10: LOC_Os08g06280 and P11: LOC_Os10g39640. (e) Correlation of the expression level data from RNA-seq and qPCR. Pearson correlation coefficient values are shown at the top left corner of each plot.

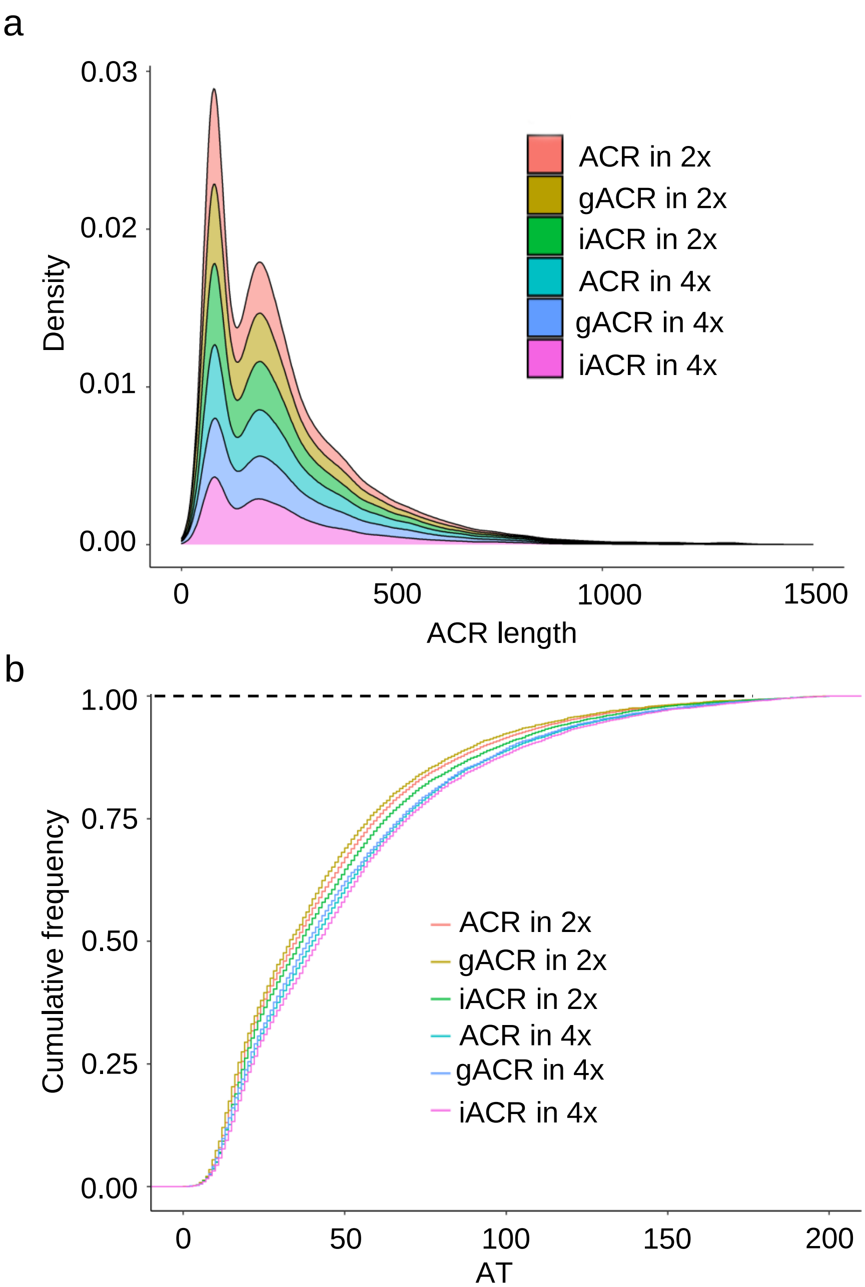

Figure S5. Comparisons of properties of ACRs in wild-type and autotetraploid rice leaves.
(**a**) Comparison of transcript lengths of different types of ACRs from wild-type and autotetraploid rice leaves. (**b**) AT content of three types of ACRs in the 2x and 4x rice genomes.

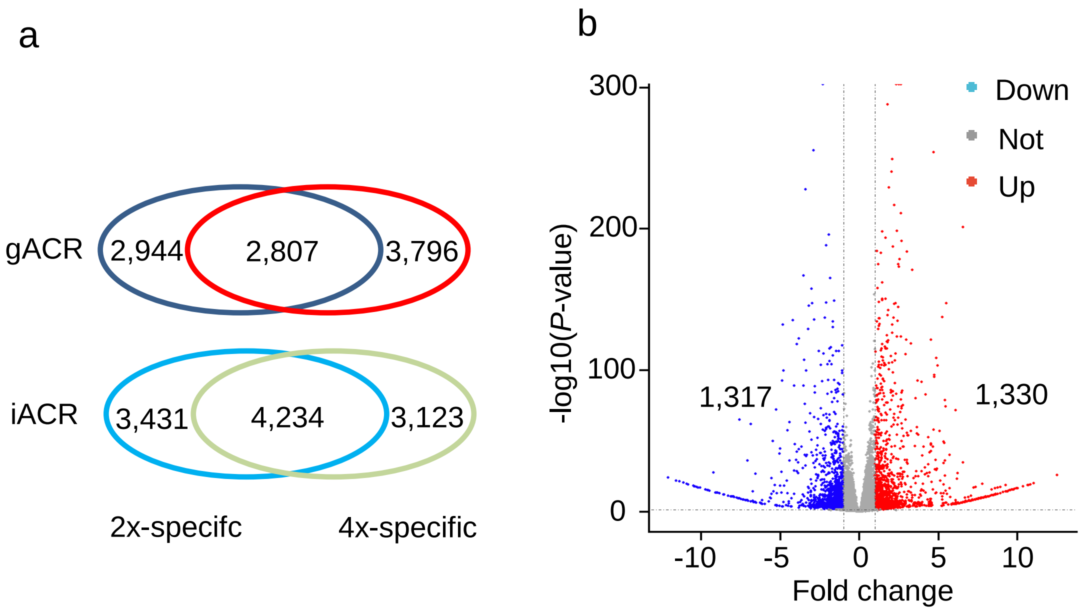

Figure S6. Functional analysis of DAGs associated with specific gACRs and iACRs in diploid and autotetraploid rice.

(**a**) Venn diagram of gACR- and iACR-associated genes in 2x and 4x rice. (**b**) Total numbers of differentially expressed genes (FC > 2, *P*-value < 0.05) in autotetraploid rice compared to wild type.

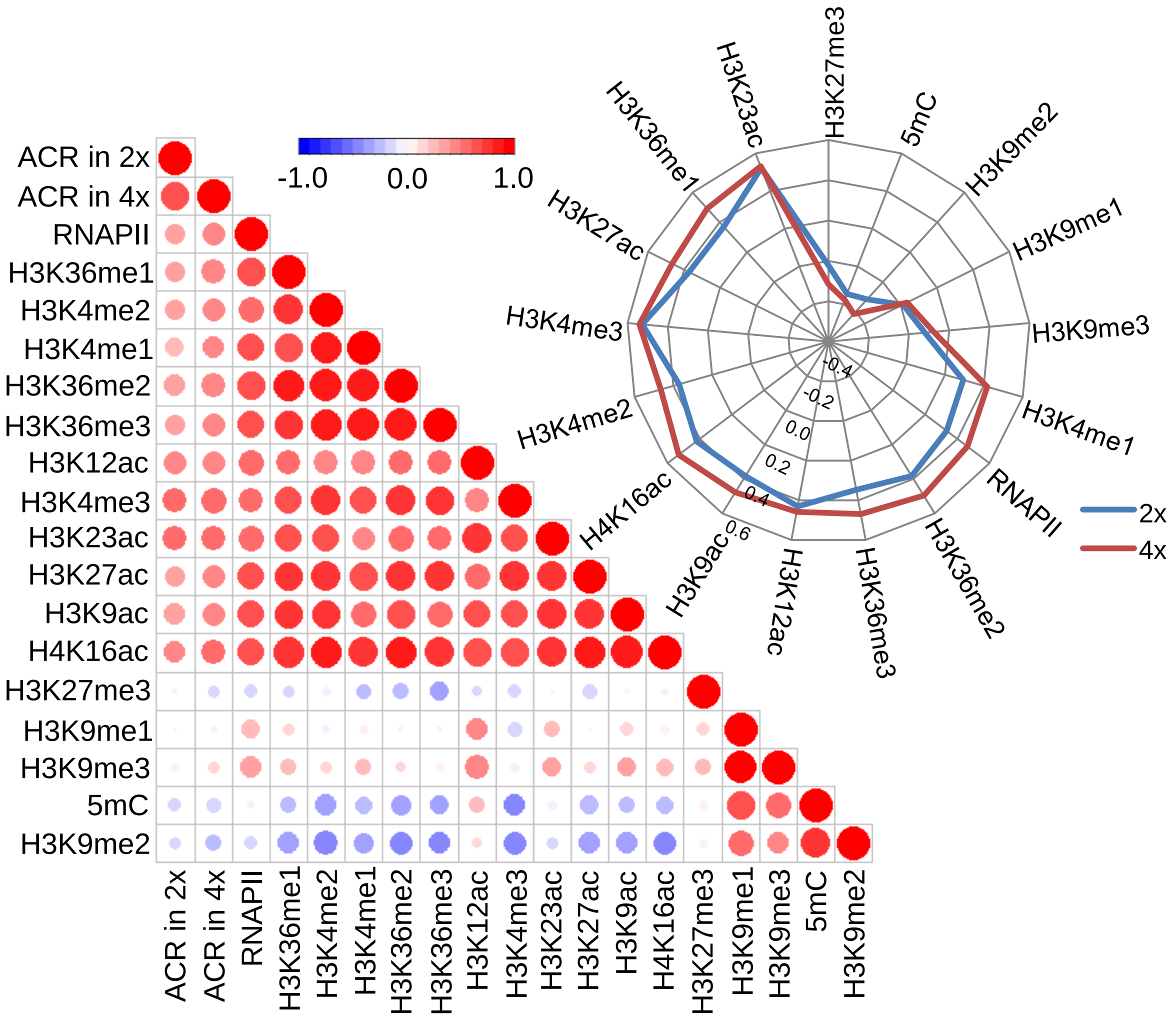

Figure S7. Correlogram of ACRs in 2x and 4x rice, as well as other epi-marks.

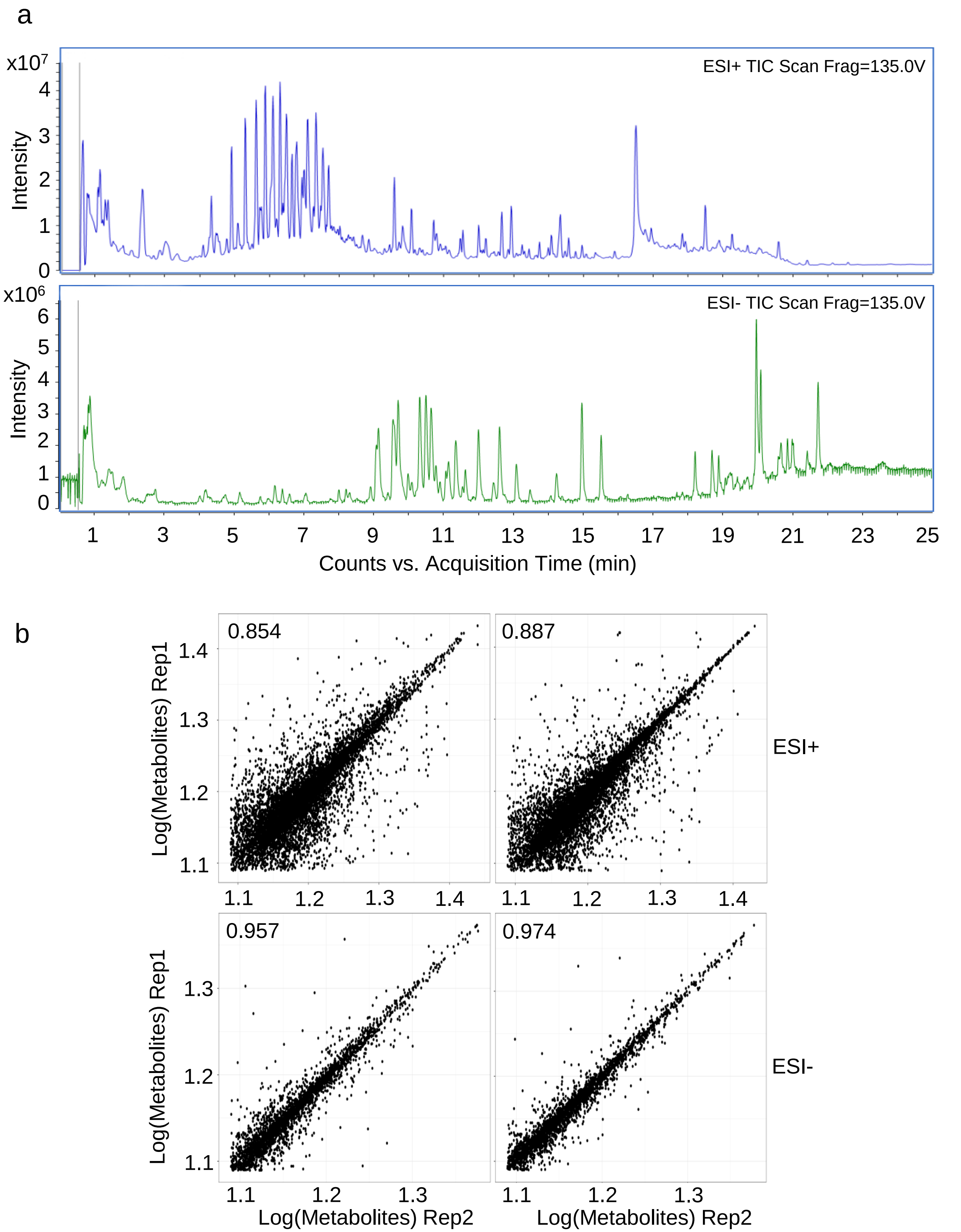

Figure S8. Quality analysis of metabolite replicates.
(**a**) Total ion current chromatograms of diploid and autotetraploid rice leaf extracts in positive and negative ESI modes. (**b**) Scatter plots of the accumulation of metabolites detected from two replicates of 2x and 4x rice leaves. Rep1 and Rep2 are two biologically independent experiments. The values ranging are plotted on both axes. Pearson correlation coefficient values are shown in the top left corner of each plot.

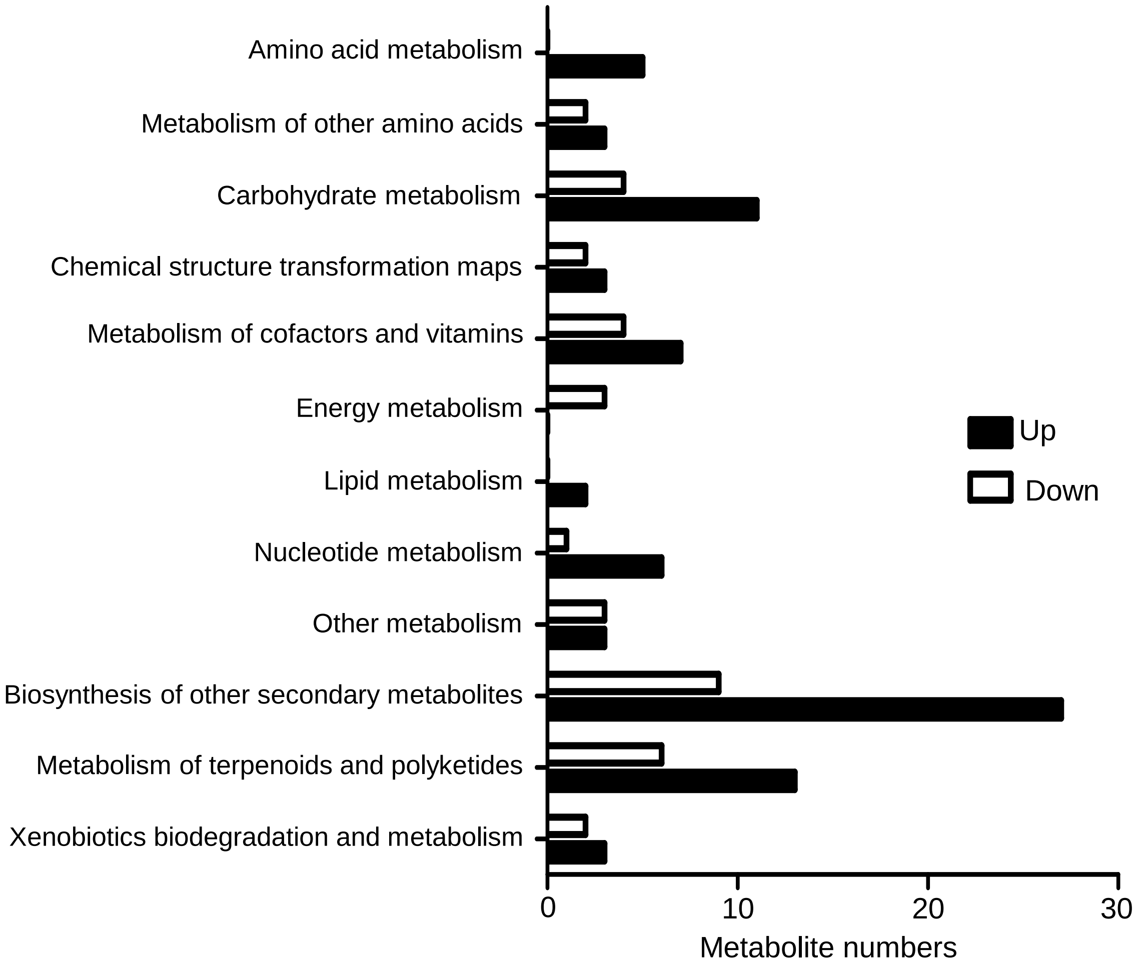

Figure S9. Numbers of DAMs in diploid and autotetraploid rice.
